# THA in patients with neglected acetabular fractures

**DOI:** 10.1051/sicotj/2022028

**Published:** 2022-08-26

**Authors:** Ashish Singh, Kartheek Telagareddy, Purushotam Kumar, Sushil Singh, Rabindra Narain Singh, Pankaj Kumar Singh

**Affiliations:** Anup Institute of Orthopaedics and Rehabilitation Patna 800020 Bihar India

**Keywords:** Neglected acetabular fracture, Total hip replacement, Protusio, Impaction grafting

## Abstract

*Introduction*: Total hip arthroplasty (THA) outcomes in patients with neglected acetabular fractures are less favourable compared to THA for osteoarthritis or inflammatory arthritis. These poorer clinical outcomes are largely due to an unexpected bone deficiency, and the procedure is more time-consuming and complicated for cases that require acetabular reconstruction and bone grafting. The clinical outcomes of THA in neglected acetabular fractures are not often studied. *Methods*: This study is a retrospective single surgeon series of THA for 51 neglected acetabular fractures in 49 patients treated with THA alone, open reduction and internal fixation with THA, or acetabular defect reconstruction THA. Our series aims to focus on the surgical technique and describe the functional and radiological outcomes of neglected acetabular fractures treated with different THA approaches by a single surgeon. *Results*: Using the Harris Hip score at the mean long-term follow-up, there was a clear improvement in 90% of patients in the present study. The long-term results are encouraging in terms of clinical improvement, radiographic restoration of acetabular bone stock, and the centre of restoration. *Discussion*: The series shows that with proper planning and reconstruction using structural bone grafting techniques, a neglected fracture-dislocation with loss of structural support can be satisfactorily treated using primary components alone. Bony reconstruction and use of primary cementless components ensure long-term survival and preserve bone stock for an easier revision of THA, if necessary, in the future.

## Introduction

**Figure 1:**
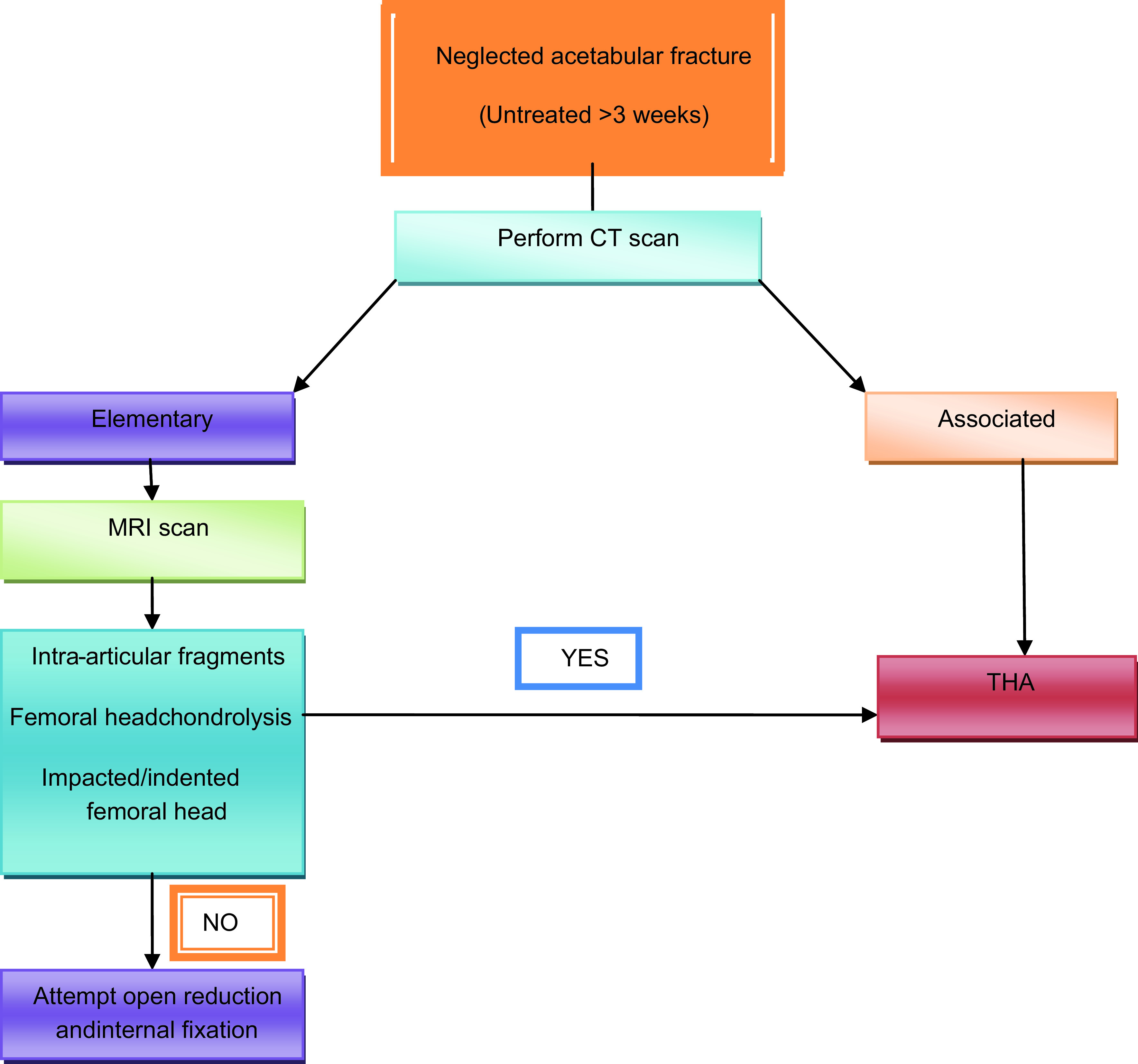
Recommended treatment approach for neglected acetabular fractures.

Acute acetabular fractures result from significant trauma, and the anatomic location and complexity of the three-dimensional structures make their treatment extremely challenging. The spectrum of treatment spans from conservative methods to percutaneous fixation to open reduction and internal fixation (ORIF). An acetabular fracture is considered neglected if the injury occurred more than 3 weeks prior [1]. Total hip arthroplasty (THA) is generally reserved for patients with acetabular fracture complications, mainly secondary arthritis and avascular necrosis of the femoral head. However, the results following ORIF for complex neglected acetabular fractures are predictably poorer due to excessive callus formation and the consequences of non-or mal-united acetabulum fragments, such as early post-traumatic arthritis, that negatively impact patient the quality of life [2]. ORIF is typically performed for simple fractures, but it is sensible to perform magnetic resonance imaging to exclude patients with conditions that can contribute to secondary osteoarthritis, such as intra-articular fragments, chondrolysis, and femoral head indentation/impaction. In these cases, ORIF alone will not improve the patient’s quality of life, and primary THA can be advantageous in these situations (Figure 1).

THA outcomes in patients with neglected acetabular fractures are less favourable compared to THA for osteoarthritis or inflammatory arthritis [3]. These poorer clinical outcomes are largely due to an unexpected bone deficiency, and the procedure is more time-consuming and complicated for cases that require acetabular reconstruction and bone grafting. Here we share our centre’s experience performing THA for 51 neglected acetabular fractures treated with THA alone, ORIF with THA, or acetabular defect reconstruction with THA. Our series aims to focus on the surgical technique and describe the functional and radiological outcomes of neglected acetabular fractures treated with different THA approaches by a single surgeon.

## Methods

### Patients

**Figure 2:**
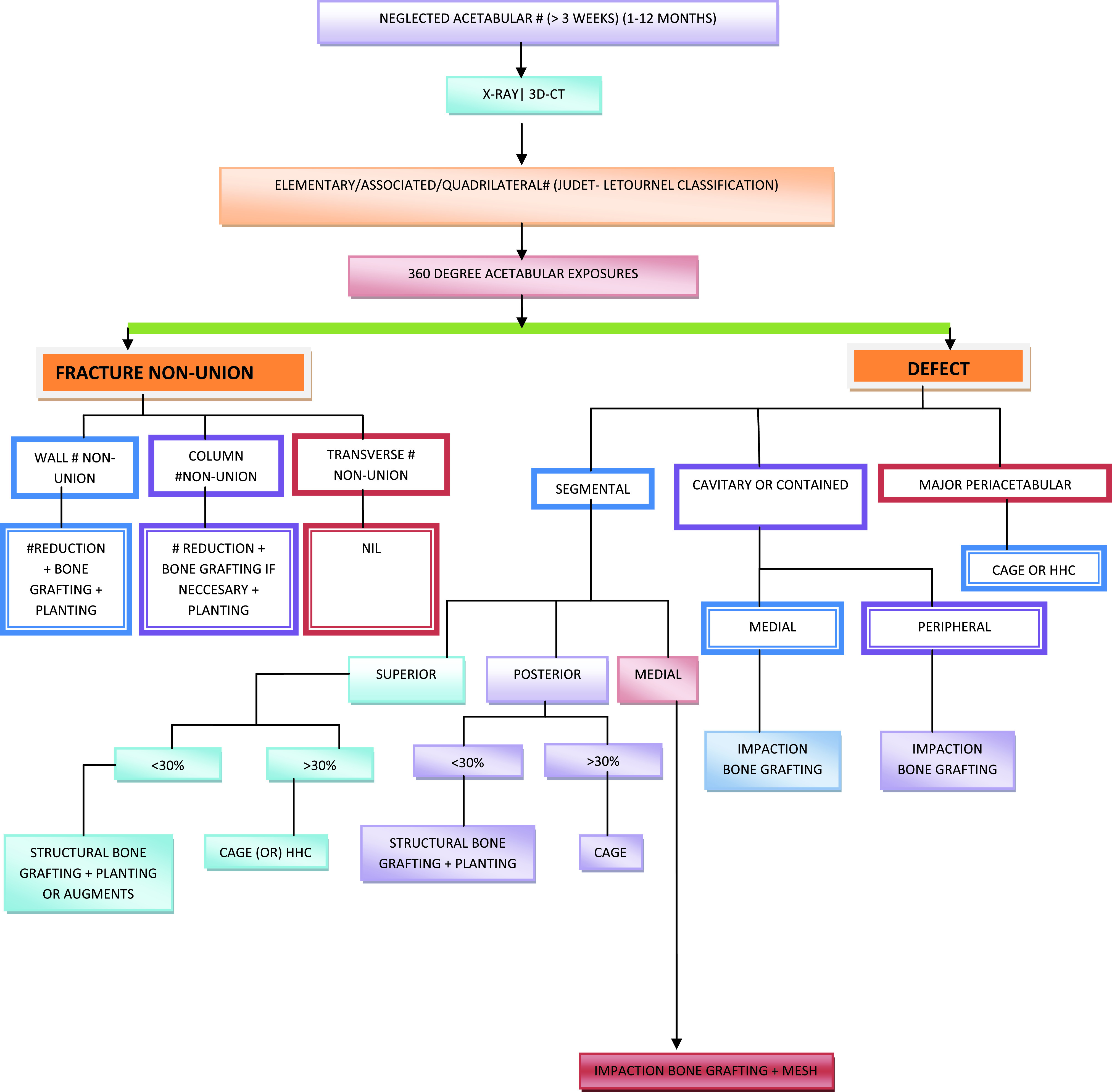
Overview of management of neglected acetabular fracture at our institute.

The records of patients who underwent THA for neglected acetabular fracture of the hip from 2014 to 2018 were reviewed. There were 41 males and 8 females (two patients had bilateral hip fractures) with a mean age of 49 years (range 25–80) at the time of surgery (Table 1). Fractures were classified according to the Judet and Letournel system using radiographs and computed tomography scans. There were 19 elementary (simple) and 32 associated (complex) fracture patterns. After exposing the acetabulum intra-operatively, the fractures were assessed and categorised as non-union and/or defects. Based on this assessment, one or more of the following strategies for defect reconstruction were considered: impaction grafting, structural grafting, mesh, or an augment (Figure 2). The cup fixation method was cemented in 6 patients and cementless in 45 patients (Table 2).

**Table 1:** Patient demographics (*n* = 49).

Mean age	48.2
Sex (male/female)	41/8
Mean BMI	26
Mode of injury	Road traffic accident
Mean time between trauma and surgery	6.7 months
Side(right/left)	35/16
Bilateral Hip	2
Pre-operative neurological status	
Foot drop	2

Abbreviations: BMI, body mass index.

**Table 2:** Use of cemented and uncemented cup with coating.

Cup size (minimum–maximum)	44–58 mm
Un-cemented	45 hips
Cemented (×3 rimfit)	6 hips
Implant	
Gription (porous coating)	25
Zimmer trilogy (trabecular metal)	6
Stryker trident (hydroxyapatite coating)	14

Outcomes were classified as excellent/good or failed/poor based on the Harris Hip Score (HHS, Table 3). Since treatment was standard, data were further analysed to assess the influences of age, sex, time interval between injury and presentation, follow-up duration, sciatic nerve lesion on admission, and mechanism of injury.

**Table 3:** Post-operative HHS scores by injury type.

	HHS score (mean value)						
	Pre-op	Post-op	6 weeks	3 months	1 year	2 years	5 years
Fracture non-union							
Fracture non-union							
Wall fracture non-union (*n* = 10)	0	12.2	42.5	68.4	84.6	95	99
Segmentation							
Defect							
Superior							
<30%	0	14	70	80	81.5	95	99.3
>30% (*n* = 0)							
Posterior							
<30%	0	15	75	82	82	90	95
>30%	0	13	40	65	74	85	95
Cavitary							
Medial							
Medial (*n* = 17)	22.1	20	45	68	85	93	97
Peripheral (*n* = 2)	15	15	45	67	84	95	98
Major periacetabular fracture (*n* = 4)	0	10	30	55	75	88	95

All patients were assessed at 6 weeks, 6 months, 1 year, 2 years, and 5 years after surgery. Post-operative radiographs were analysed in terms of fracture healing and implant loosening. Radiographic failure was defined as radiolucent lines in all three zones, according to DeLee and Charnley [4]. Clinical failure was defined as the need for acetabular component revision for any reason.

During the post-operative period, the patient’s received deep venous thrombosis prophylaxis, appropriate systemic antibiotics, and indomethacin to prevent heterotrophic ossification (HO) per our centre’s protocol. Mobilisation was individualised according to the reconstructive procedure performed. If we did not reconstruct the acetabulum or use a cemented cup with impaction grafting, patients were mobilised within 24 h with full weight-bearing. In other cases, non-weight bearing with a walking aid was advised for the first six weeks. Weight-bearing gradually increased, and patients performed unassisted mobilisation after reviewing the radiographs and as circumstances allowed.

### Surgical technique

The patient is positioned in the lateral decubitus position with appropriate padding of pressure areas. We prefer the standard postero-lateral approach because it allows posterior column reduction, plating, and bone grafting with simultaneous THA. After dividing the fascia lata, the gluteus maximus is split along the length of the fibres. The sciatic nerve is identified and carefully mobilised from the scar tissue. The hip is dislocated, and the femoral neck is resected. The head is preserved as an autograft if the bone is of reasonable quality. The ischial spine is identified if posterior column fixation is necessary.

**Figure 3:**
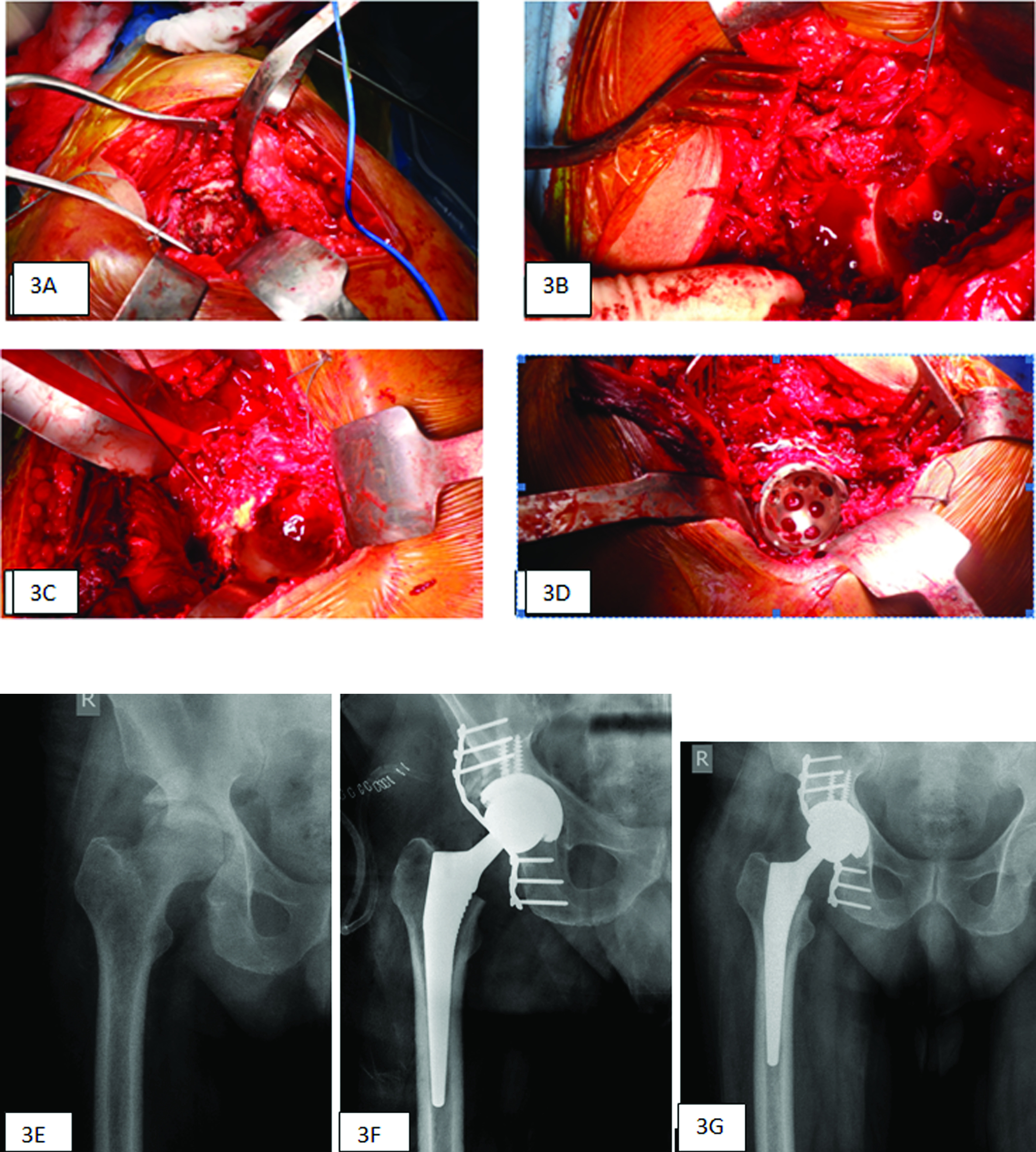
(A) 360° view of a neglected acetabular fracture and analysing the defect intra-operatively. (B) Posterior wall defect. (C) Provisional fixation of the defect with K-wires. (D) Final fixation with plate and cup. (E) Radiograph showing a posterosuperior wall acetabulum fracture. (F) Immediate post-operative radiograph showing fracture reduction and fixation with a plate. (G) Radiograph at 5-years follow-up.

The acetabulum is then fully exposed (Figure 3A) to enable assessment of the fracture area and bone loss. Meticulous examination of the acetabular floor and wall is done to identify all defects. Special care is taken to locate the transverse acetabular ligament at the inferior aspect of the acetabulum. The reconstruction is built up from this level to restore hip mechanics. All fibrous tissue, scar tissue, and necrotic bone are debrided. The anterior column is also inspected. If both columns are fractured with extensive comminution in the pre-operative assessment, we prefer to wait for some healing to occur over 6–12 weeks post-injury before performing THA. This allows the comminuted fragments – particularly those medially and anteriorly – to heal and makes the technical reconstruction easier.

### Non-union fracture reduction and stabilisation

Our basic aim is to preserve the host bone and soft tissues of the fractured fragment of the posterior wall and maximally mobilise the column using standard reduction techniques. The fibrous and scar tissues within the fracture are excised to mobilise the fragments. Once the fracture is provisionally reduced, it is fixed with Kirshnerwires (Figures 3B and 3C) and later reinforced with a plate along with morselized bone graft. Although the anatomic reduction is not essential, major deformity correction and stability are required to optimise cup implantation (Figures 3D–3G). For mildly displaced non-unions, bone grafting with autograft is performed in the freshly debrided defect prior to fixation. If non-unions are widely separated, we prefer to mobilise, reduce, re-align, and re-enforce them prior to bone grafting to improve the deformity and restore the anatomy. Acetabular defect analysis is performed after fixation and defined accordingly. Once the columns are stably fixed, and there is no segmental defect after fractured fragment reduction, the acetabulum is reamed to fit the definitive acetabular component. Considering bone conservation, reaming is restricted to a bleeding surface.

### Defect reconstruction

Various surgical techniques can be used for defect management. Small contained and cavitary defects can be addressed with impaction grafting using autografts. Large peripheral segmental defects can be managed either by structural autografts from the femoral head or trabecular metal augments. We prefer to use structural bone grafts with a combination of morselized grafts for segmental defects and morselized bone grafts for cavitary defects.

### Dealing with central/floor bone defects

**Figure 4:**
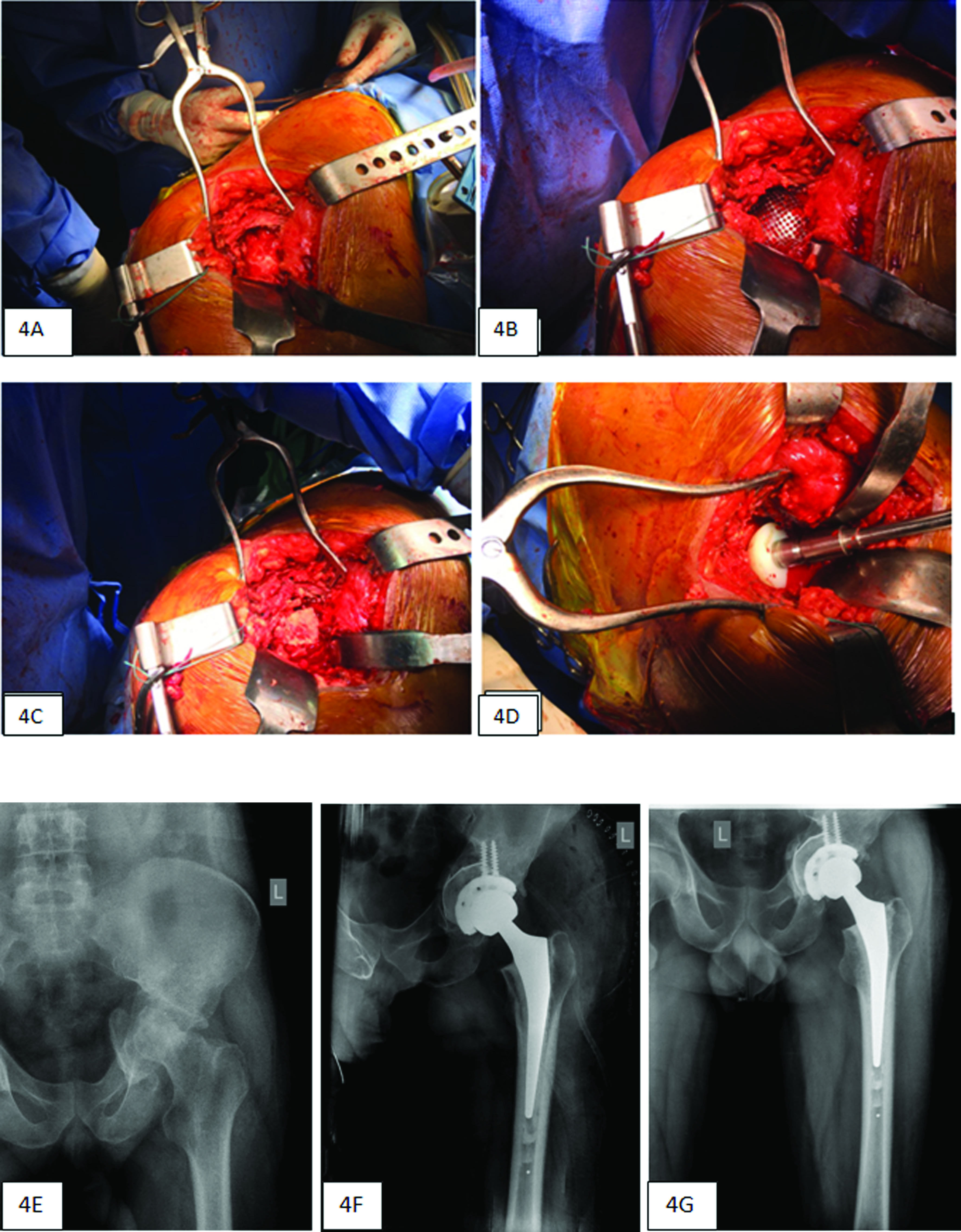
(A) Central defect with no intact medial wall. (B) Reconstruction of the defect with mesh and bone graft. (C) Bone graft over the mesh. (D) Impaction. (E) Radiograph showing a medial wall defect. (F) Radiograph showing medial wall reconstruction with mesh and bone graft. (G) Well-consolidated bone graft at 5-years follow-up.

These defects usually arise from fractures involving the quadrilateral plate. When the medial wall is intact, we use impaction grafting; for medial wall defects, we prefer mesh and impaction grafting, both in cemented and cementless cups (Figures 4A–4E).

The use of small and large impactors is indispensable to achieving graft stability. Care is taken to reconstruct hip anatomy by packing as much chip graft material as necessary until the socket is brought down to the level of the transverse ligament. After impaction, the pre-existing enlarged acetabular diameter decreases, and this can be secured for cup implantation.

### Dealing with posterosuperior segmental bone loss

**Figure 5:**
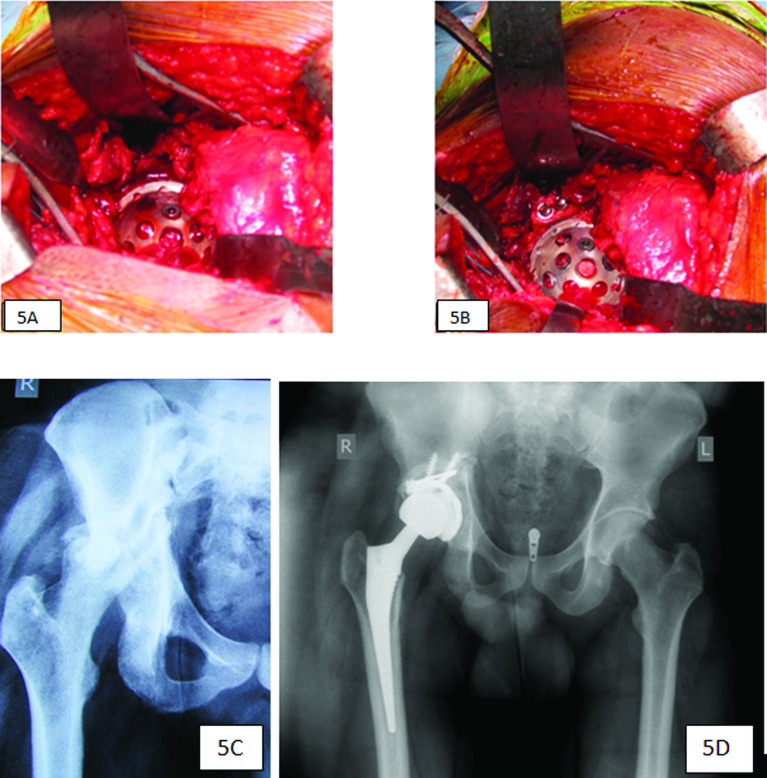
(A) Superior wall defect. (B) Superior defect reconstruction with a structural bone graft and screw fixation. (C) Radiograph showing a superior wall fracture with femoral head subluxation. (D) Radiograph showing good bone graft incorporation at five years.

In these cases, the displaced posterosuperior fragment of the acetabular wall is usually held by the scar tissue. The bone piece is usually osteoporotic and often crushed. If there are good soft tissue attachments, it may be possible to incorporate the remnant during reconstruction. If the piece is crushed or necrotic, it is removed and reconstructed. Our preference is to use femoral head autografts. For an isolated superior segmental defect, screw fixation is sufficient. The acetabular bed is freshened using reamers. The femoral head autograft can be debulked and shaped to match the corresponding surface of the host acetabulum, where it is held with provisional Kirschner wires. Definitive fixation can be achieved with screws (Figures 5A–5C).

However, posterior defects will be subjected to significant mechanical forces during patient mobilisation, and such defects need femoral head autografts stabilised with a posterior column plate and screws in the directions of forces. When the column is additionally fractured, the plate can be contoured to stabilise the columns. If the defect is quite posterior, we prefer to use a posterior column plate to support and buttress the posterior surface of the structural femoral bone graft. Following adequate femoral graft fixation, it is shaped using reamers, and the acetabulum is prepared for the cup implantation. In major peri-acetabular defects, when the cup placement is not stabilised at the anatomic centre, proximal cup placement achieves stability.

## Results

The operative duration was 2–3 h with 850–1500 mL of blood loss. The HHS increased from a pre-operative mean of 9.2 to a post-operative mean of 42.6 at 6 weeks, 69.3 at 3 months, 83.3 at 1 year, and 93 at 2- and 5-years. Overall, 45 patients (88.2%) had an HHS of good to excellent (>80) at the most recent follow-up. Complications in this series included HO in one (1.9%) patient and aseptic loosening in four (7.8%) patients (Table 3). Based on the Judet and Letournel classification for loosening, three patients had the associated type, and one was the elementary type. In all remaining cases, the grafts appeared to be well incorporated at the final radiographic follow-up at 5 years. Most grafts showed a characteristic increase and then decreased in density with subsequent trabecular patterning by 3-months. After formation, none of these regions showed further loss of density or trabeculae in the grafts.

## Limitation of this study

In this study, we have done magnetic resonance imaging studies to see Intra-articular fragments, femoral head chondrolysis, and impacted/indented femoral head, and if viable, we have attempted an osteosynthesis in place of total hip replacement.

## Discussion

The complexity of this kind of neglected fracture makes the treatment extremely challenging as the problems become magnified and makes surgeons more focused on the type of fracture presentation either is neglected or fixed with percutaneous fixation or open reduction and fixation and converting into a total hip replacement to give optimal functional outcomes.

This was a single surgeon series of 51 hips that underwent THA for neglected acetabular fractures. They were managed with different acetabular reconstruction techniques to achieve stable cup placement. Selecting an appropriate surgical technique to achieve stability depends on the type, size, and location of the acetabular fracture and the defect if it is present. In this series, 88.2% of patients had good to excellent outcomes at the end of the 5-year follow-up, which indicates that the reconstruction technique was successful in a neglected acetabular fracture where we expect to find defects during the surgery.

We hypothesised that stable cup placement and acetabular bone conservation allows use of the primary hip component; this improves prosthesis longevity by osteointegration and predicts good results. Poor initial stability and cage use may have increased the risk of later revision.

Non-unions are debrided, freshened to bleeding bone, and treated with stable fixation with posterior column plating and bone grafting. Some authors have expressed concerns that the application of a plate to the posterior column in the setting of non-union may result in osteonecrosis of the fragment [5]. Major structural bone grafts may be resorbed, and implants can break or loosen if the fracture does not unite. Concerns have been raised that mobilisation of the displaced bony fracture fragment could interrupt blood supply. In our series, all patients had good results after management with fracture reduction, stabilisation, and re-enforcement with a plate along with morselized bone graft. This might be due to the meticulous handling of the soft tissue attachments of the bony fragments. Out of 10 patients, only 2 had aseptic loosening with this technique.

For segmental bone loss, the common practice of using bone grafts to fill the acetabular defect needs to be critically looked at. Uncemented porous-coated acetabular cups are not expected to gain bone in-growth from areas in which they are exclusively in contact with bone grafts (two dead surfaces cannot anchor a living process). In these areas, a fibrous membrane usually forms between the graft and uncemented implant [6], and this is not expected to reliably provide long-term implant fixation. Haaren et al. previously reported a high failure rate of impaction grafting in large acetabular defects [7]. When >30% of the cup was supported by graft, there was a significantly higher risk of failure. In our series, three patients had large defects in the superior wall. Harris et al. [8] reconstructed such defects with a structural bone graft from the femoral head with screws, whereas we used metal superior augmentation in one patient that was subsequently revised due to aseptic loosening. We had used a structural bone graft from the femoral head for the reconstruction of the posterior wall in one patient with a >30% defect, but it eventually failed due to bone graft absorption. Despite its widespread use and the favourable outcomes reported by some authors, much is still unknown about the biological fate of these bone grafts due to limited data.

Specialised bi-lobed or oblong cups have been used for superolateral defects involving a wall segment, but we have no experience with these. Pelvic reinforcement cages/rings have been described in the literature for unstable non-unions pelvic discontinuity, and major structural defects.

In this series, 17 patients with central defects with intact medial walls were managed with impaction grafting, and 6 patients without intact medial walls were managed with impaction bone grafting plus flexible stainless-steel mesh. Ranawat and Zahn [9] recommended that bone grafting is not required in cases in which the protrusion is <5 mm. When the protrusion is >5 mm, and there is an intact medial wall, bone grafting without an augmentation device is appropriate. If there is a gross deficiency of the medial wall, a bone graft possibly with additional fixation devices (mesh, central augments) should be considered. Among 17 patients, only one had aseptic loosening. The biologic reconstruction method with impaction grafting and cementless fixation has a high success rate, and various reports on this technique agree with each other. Mullaji and Marawar [10] also reported good to excellent outcomes after impaction grafting with protrusions.

In two patients with bilateral major peri-acetabular defects, we could not stabilise the cup at the anatomical position of the acetabulum. These were managed with the high hip centre technique to avoid using a cage. Proximal placement of the hip centre increases contact between host bone and the implant. It also reduces the need for cages in cases with severe bony deficiency, while the majority of the reamed host bone is superior to the anatomic hip centre. A high hip centre technique was reported by Schetzer and Harris [11], and their results without cage use were encouraging. The bilateral high hip centre technique significantly reduces the operation time and provides faster post-operative rehabilitation; it also results in gait parameters similar to those with bilateral anatomical reconstruction, regardless of hip centre placement [12]. Although we believe that the biomechanical advantages of normal anatomy should be considered during surgical technique selection, in cases where the condition dictates (e.g., peri-acetabular bone insufficiency), it is also possible to use a high hip centre if it is to be applied bilaterally.

Using the HHS at the mean long-term follow-up, there was a clear improvement in 90% of patients in the present study. The long-term results are encouraging in terms of clinical improvement, radiographic restoration of acetabular bone stock, and the centre of restoration. However, despite overall good mid-term outcomes (~5–7 years), there is no consensus regarding which reconstruction technique guarantees better long-term survivorship (~10 years) due to the lack of high-quality, long-term studies on modern reconstructive options. Finally, the outcome of complex THA depends on multiple factors like surgeon expertise, implant availability, evaluation of the bone defects, meticulous planning, and good post-operative care and rehabilitation. The strength of this study is that a single surgeon performed all operations with mid-to-long-term follow-up in complex neglected acetabular fractures. The limitations are the small sample size and no long-term outcomes (>10 years). The series shows that with proper planning and reconstruction using structural bone grafting techniques, a neglected fracture-dislocation with loss of structural support can be satisfactorily treated using primary components alone. Bony reconstruction and use of primary cementless components ensure long-term survival and preserve bone stock for an easier revision THA, if necessary, in the future.

The results of our case series suggest that THA might be an effective treatment for neglected acetabular fractures. One critical challenge is how to approach the acetabular defect to obtain a stable hip. Neglected associated fracture types are more difficult to repair and have poorer surgical outcomes compared to elementary types. Successful outcomes are expected if a solid bone stock is achieved using various surgical techniques.
